# The relation of recurrent laryngeal nerve to inferior thyroid artery and extralaryngeal nerve branching may increase the risk of vocal cord paralysis in thyroidectomy

**DOI:** 10.1007/s00423-024-03392-y

**Published:** 2024-06-27

**Authors:** Nurcihan Aygun, Mehmet Taner Unlu, Ozan Caliskan, Mehmet Kostek, Adnan Isgor, Mehmet Uludag

**Affiliations:** 1 Department of General Surgery, University of Health Sciences, Sisli Hamidiye Etfal Training and Research Hospital, Huzur Avenue, Cumhuriyet Street, Sariyer, Istanbul, 34371 Turkey; 2 Department of General Surgery, Sisli Memorial Hospital, Istanbul, Turkey

**Keywords:** Vocal cord paralysis, Recurrent laryngeal nerve, Inferior thyroid artery, Extralaryngeal nerve branching, Thyroidectomy

## Abstract

**Purpose:**

The anatomical variations of the recurrent laryngeal nerve (RLN) are common during thyroidectomy. We aimed to evaluate the risk of RLN paralysis in case of its anatomical variations, retrospectively.

**Methods:**

The patients with primary thyroidectomy between January 2016 and December 2019 were enrolled. The effect of age, gender, surgical intervention, neuromonitorisation type, central neck dissection, postoperative diagnosis, neck side, extralaryngeal branching, non-RLN, relation of RLN to inferior thyroid artery (ITA), grade of Zuckerkandl tubercle on vocal cord paralysis (VCP) were investigated.

**Results:**

This study enrolled 1070 neck sides. The extralaryngeal branching rate was 35.5%. 45.9% of RLNs were anterior and 44.5% were posterior to the ITA, and 9.6% were crossing between the branches of the ITA. The rate of total VCP was 4.8% (transient:4.5%, permanent: 0.3%). The rates of total and transient VCP were significantly higher in extralaryngeal branching nerves compared to nonbranching nerves (6.8% vs. 3.6%, *p* = 0.018; 6.8% vs. 3.2%, *p* = 0.006, respectively). Total VCP rates were 7.2%, 2.5%, and 2.9% in case of the RLN crossing anterior, posterior and between the branches of ITA, respectively (*p* = 0.003). The difference was also significant regarding the transient VCP rates (*p* = 0.004). Anterior crossing pattern increased the total and transient VCP rates 2.8 and 2.9 times, respectively.

**Conclusion:**

RLN crossing ITA anteriorly and RLN branching are frequent anatomical variations increasing the risk of VCP in thyroidectomy that cannot be predicted preoperatively. This study is the first one reporting that the relationship between RLN and ITA increased the risk of VCP.

## Introduction

Recurrent laryngeal nerve (RLN) injury is still among the major complications despite the increased data on the anatomy, variations, and function of the RLN, and also technical and technological advances in nerve protection in thyroidectomy. It has been reported that many clinical scenarios such as thyroid cancer, neck dissection, Graves’ disease, thyroiditis, enlarged goiter, recurrent benign and malignant diseases, total resection of the thyroid lobe, failure to identify RLN, reoperation for postoperative bleeding, low-moderate hospital volume, low surgeon volume, extralaryngeal branching of the RLN, and non-recurrent laryngeal nerve (NRLN) are associated with the potential risk of post-thyroidectomy vocal cord paralysis (VCP) [1–6].

Anatomical variations of RLN are usually preoperative unpredictable risk factors, even though factors such as diagnosis, type of intervention, hospital volume, and surgeon’s experience are preoperative predictable risk factors [5]. Anatomical variations of RLN, such as extralaryngeal branching of RLN, distorted RLN, the relation of inferior thyroid artery (ITA) or its branches with RLN, and NRLN may be the potential cause of nerve injury due to visual misidentification or difficulty in detecting the nerve [7]. Although NRLN is a rare anatomical variation, it has been reported to increase the risk of VCP [8]. Extralaryngeal branching is a frequent variation and it has been shown to increase the risk of RLN paralysis, even though VCP rates were found to be similar in branching and non-branching nerves in some studies [9–12].

The relationship between RLN and ITA has been evaluated in many studies [13]. The effect of the relationship between ITA and RLN on the risk of VCP has been evaluated only in one study and no significant effect has been detected even though it has been claimed in anatomy studies that the risk of RLN injury was higher when RLN was located in the anterior or between the branches of the ITA [12, 14, 15].

The present study aims to evaluate the risk of VCP in anatomical variations of RLN, particularly considering the relationship between ITA and RLN.

## Materials and methods

### Ethical review and study design

This study was approved by the local ethics committee (no:3142, approval date: 2/16/2021) and the data of patients who underwent thyroidectomy (+ parathyroidectomy) with intraoperative neural monitoring (IONM) were evaluated retrospectively in accordance with the guidelines in the Helsinki Declaration. Each operated neck side was considered a separate entity. Anatomical variations of RLN and its relationship with the ITA were investigated in these patients and their relationship with VCP was evaluated.

### Study population

The nerves of the patients, which were exposed till the laryngeal entry point, between January 2016 and December 2019, were included in the study. After lobectomy, detection of malignancy in the pathology, the completion thyroidectomy side was also considered as the primary intervention.

**Fig. 1:**
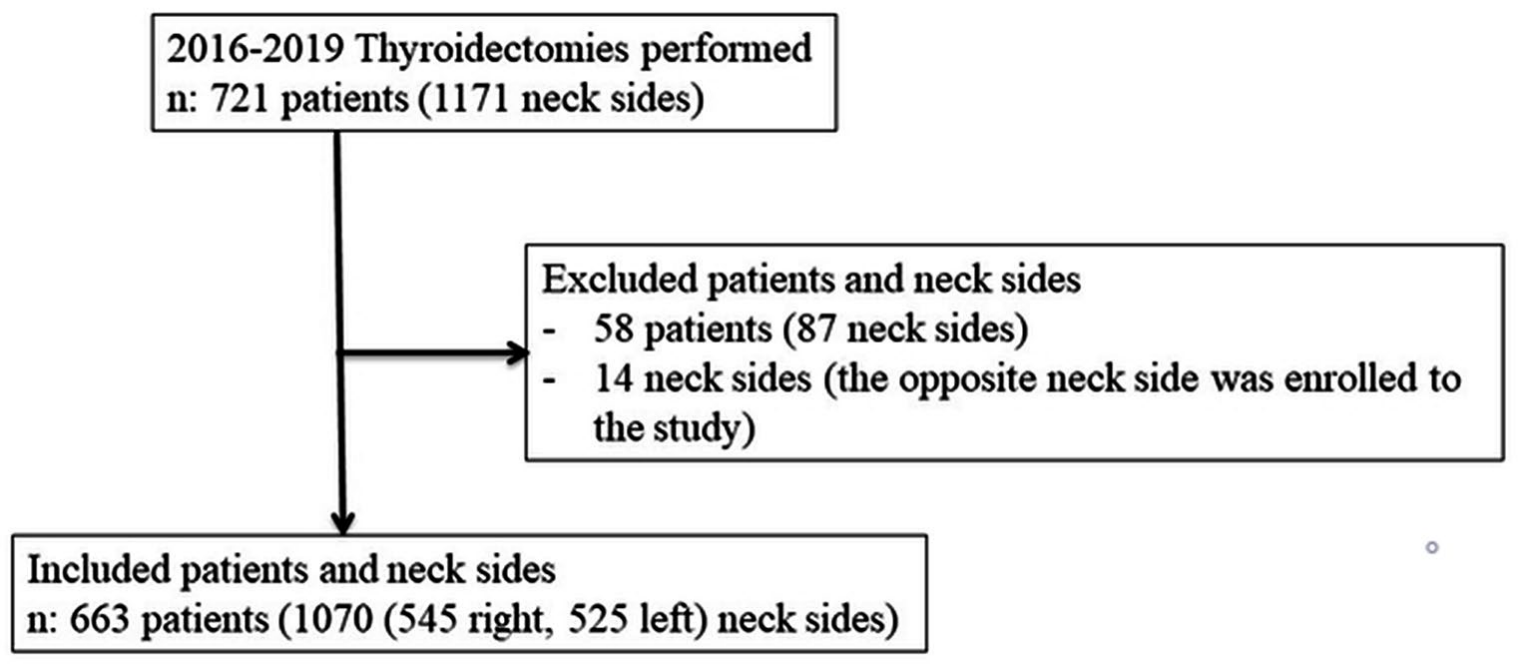
Flow-chart of study

Patients were excluded based on the following criteria: Patients who underwent secondary thyroidectomy, those with preoperative VCP and resected RLN due to tumor invasion, parathyroid or subtotal thyroidectomy procedures where RLN course was not fully exposed, and those with incomplete recording of the RLN anatomical variations and without postoperative vocal cord examination were excluded from the study (Fig. 1).

### Variables

Gender, age, surgery performed, type of neuromonitoring, postoperative diagnosis, pre- and postoperative vocal cord examination, nerve side, extralaryngeal nerve branching, NRLN, RLN and ITA relationship, grade of the Zuckerkandl tubercle were evaluated.

RLN was defined as extralaryngeal branching if it branched > 5 mm before entering the larynx and all its branches entered the larynx [10].

Zuckerkandl tubercles were divided into 4 grades according to their size. If not seen, it was defined as grade 0; if there was only the thickening of the lateral edge of the thyroid lobe, as grade 1; if < 1 cm, as grade 2; and if > 1 cm, as grade 3 [16].

Preoperative and postoperative vocal cord examinations were performed by an independent otolaryngologist. Periodic vocal cord examination of patients with VCP was performed on postoperative 15th day and at months 1, 2, 4, and 6. It was defined as temporary if the VCP improved in 6 months, and as permanent VCP if it persisted for 6 months.

### IONM and thyroidectomy

All operations were performed with low dose neuromuscular agent (rocuronium 0.3 mg/kg) under general anesthesia and with intermittent or continuous IONM. IONM was performed using NIM 3.0 Nerve Monitoring system (Medtronic, Jacksonville, FL) with an endotracheal tube with surface electrodes. The installation, implementation, and data evaluation of IONM were evaluated in accordance with the guidelines of the International Nerve Monitoring Study Group [17].

The thyroid region was entered through the strap muscles with an anterior approach. In bilateral interventions, surgery was started from the side having a pathology requiring surgical indication. Staged thyroidectomy was performed in patients with a signal loss on the first side. RLN was generally identified at the level of ITA. RLN was identified close to its laryngeal entry with a superior approach in large substernal goiters. After the RLN was identified, the thyroid lobe was dissected gradually by separating the tertiary branches of the ITA on the thyroid capsule at the anteromedial side of the RLN. RLN was exposed from its identification point to the entry of the larynx or its cervical course was fully exposed to define its extralaryngeal branching and relationship with ITA.

### Statistical analysis

The data were evaluated using IBM SPSS Statistics version 25 (IBM, Armonk, NY, USA). Data were presented as mean *±* standard deviation (SD) and range. In the statistical analysis, *p*-value less than 0.05 was considered as the criteria for significant results.The normal distribution of the data was tested with Kolmogorov-Smirnov test and appropriate non-parametric tests were selected. Pearson’s chi-squared and Fisher’s exact tests were used to compare categorical independent groups and the odds ratio was calculated in 2 × 2 tables for significant differences. Bonferroni Corection was applied on comparisons with more than 2 independent categorical variables. Risk calculations were shown with Odds Ratio and 95% Confidence Interval. Continuous numeric variables were compared between two independent groups by using Mann-Whitney U test and Kruskal-Wallis test was used when more than two independent groups were compared. Bonferroni correction was applied in subgroup analysis. The variable with a *p*-value less than 0.05 in univariate analysis was considered as significant and moved to multi-variable analysis. Binary logistic regression analysis was preferred for multi-variable analysis and was evaluated to determine the independent risk factors affecting VCP. The results of the analysis were evaluated by using Odds Ratio and 95% Confidence Interval.

## Results

1171 neck interventions were performed during this period (Fig. 1). After excluding 58 patients (87 neck sides) who did not meet the inclusion criteria, 677 (1084 neck sides) operations were performed in 663 patients with a mean age of 50 *±* 13.5 years (18–89). Of these patients, 14 neck sides were excluded and 1070 were evaluated in the study (Table 1; Fig. 1).

**Table 1:** Surgical procedures performed

	Patient (*n*)	Included neck side		Excluded neck side	
		Right	Left	Right	Left
TT	353	352	352	1	1
Right L + Left STL	2	2			2
TT + BCND *±* Unilateral or bilateral LND	49	49	47		2
TT + Right CND	4	4	4		
TT + Left CND	6	6	5		1
Right L + Right CND	3	3			
Left L + Left CND	5		5		
Right L	125	125			
Right L + Exploration for Left PTx	4	4			4
Left L	109		109		
Left L + Exploration for Right PTx	3		3	3	
Total	663	545	525	4	10

*TT* Total thyroidectomy, *L* Lobectomy, *STL* Subtotal lobectomy, *CND* Central neck dissection, *BCND* Bilateral central neck dissection, *LND* Lateral neck dissection, *PTx* Parathyroidectomy

The postoperative diagnosis was MNG for 632 (59%) cases, Graves’ disease for 107 (10%), and malignancy for 331 (31%), according to the neck side.

### Non-recurrent laryngeal nerve

Of the 4 NRLNs (0.37%) detected in 1070 nerves, 3 were in female patients and 1 was in a male patient. The NRLNs were all on the right side. 3 nerves had a single branch, 1 had 2 branches. Two nerves ran superior to the artery and were not associated with ITA. One nerve ran on the artery and the other between the branches of the artery. No significant difference was detected in terms of the other properties (Table 2).

**Table 2:** Comparison of the non-recurrent laryngeal nerves and recurrent laryngeal nerves regarding the clinical and anatomical features

	Nonrecurrent laryngeal nerve (*n*: 4)	Recurrent laryngeal nerve (*n*: 1066)	*p*
Age	48 *±* 7.5	49.2 *±* 13.4	0.886
BMI kg/m^2^	29.9 *±* 2.8	28.9 *±* 5.9	0.577
Gender			1
Female (*n* = 818)	3 (75%)	815 (76.5%)	
Male (*n* = 252)	1 (25%)	251 (23.5%)	
Monitorisation type			1
I-IONM (*n* = 510)	2	508 (47.7%)	
C-IONM (*n* = 560)	2	558 (52.3%)	
Intervention type			1
Tx (*n* = 959)	4 (100%)	955 (89.6%)	
Tx + CND (*n* = 111)		111 (10.4%)	
RLN and ITA relationship (n: 1062)			0.116
Anterior RLN (*n* = 487)	1 (50%)	486 (45.9%)	
Posterior RLN (*n* = 473)		473 (44.5%)	
Between the branches of ITA (*n* = 102)	1 (50%)	101 (9.6%)	
Branching type			1
Nonbranching RLN (*n* = 690)	3 (75%)	687 (64.4%)	
Branching RLN (*n* = 380)	1 (25%)	379 (35.6%)	
Branching distance (cm)	1	1.9 *±* 0.9	0.354
Weight of the thyroid lobe (g)	23.5 *±* 4.9	30.2 *±* 30.5	0.670
Grade of ZT (n:960)			0.504
Grade 0 or 1 (*n* = 570)	2	568 (59.3%)	
Grade 2 (*n* = 174)		174 (18.2%)	
Grade 3 (*n* = 216)		216 (22.5%)	

*BMI* Body mass index, *I-IONM* Intermittent intraoperative neural monitoring, *C-IONM* Continuous intraoperative neural monitoring, *Tx* Thyroidectomy, *CND* Central neck dissection, *ITA* Inferior thyroid artery, *ZT* Zuckerkandl Tubercle

### Extralaryngeal nerve branching

Extralaryngeal branching was detected in 380 (35.5%) RLNs out of 1070 (Table 3). Of the 380 branching nerves, 351 (92.4%) had 2 branches, 26 (6.8%) had 3 branches, and 3 (0.8%) had 4 branches. The rate of nerve branching was higher in females compared to males (38.5%, 25.8%, respectively, *p* < 0.0001). The branching rate was 37.8% when the nerve crossed the artery anteriorly, 30.4% when it crossed posteriorly, 49.9% when it passed through the branches, with a significant difference between the groups (*p* < 0.0001). The branching rate was 33.3%, when the grade of Zuckerkandl tubercle was 0 or 1, 43.7% when it was grade 2, and 38.4%% when it was grade 3, with a significant difference between the groups (*p* = 0.035). There was no significant difference regarding the other properties (Table 3).

**Table 3:** Comparison of branching and nonbranching nerves regarding the clinical and anatomical features

		Nonbranching RLN n: 690 (64.5%)	Branching RLN n: 380 (35.5%)	*p*
Age		49.1 *±* 13.3	49.4 *±* 13.6	0.641
BMI kg/m^2^		28.9 *±* 5.7	29 *±* 6.1	0.935
Gender				< 0.0001
Female (*n* = 818)		503 (61.5%)	315 (38.5%)	
Male (252)		187 (74.2%)	65 (25.8%)	
Monitorisation type				0.121
I-IONM (*n* = 510)		341 (66.9%)	169 (33.1%)	
C-IONM (560)		349 (62.3%)	211 (37.7%)	
Intervention type				0.766
Tx (959)		617 (64.3%)	342 (35.7%)	
Tx + CND (111)		73 (65.8%)	38 (34.2%)	
Neck side				0.143
Right (*n* = 545)		340 (62.4%)	205 (37.6%)	
Left (*n* = 525)		350 (66.7%)	175 (33.3%)	
RLN and ITA relationship (n: 1062)				0.001
Anterior RLN (n: 487)		303 (62.2%)	184 (37.8%)	
Posterior RLN (n: 473)		329 (69.6%)	144 (30.4%)	
Between the branches of ITA (n: 102)		52 (50.1%)	50 (49.9%)	
NRLN				1
(+) (*n* = 4)		3 (75%)	1 (25%)	
(−) (*n* = 1066)		687 (64.4%)	379 (35.6%)	
Branching distance (cm)			1.92 *±* 0.9	
Grade of ZT (n: 960)				0.035
Grade 0 or 1 (*n* = 570)		380 (66.7%)	190 (33.3%)	
Grade 2 (*n* = 174)		98 (56.3%)	76 (43.7%)	
Grade 3 (*n* = 216)		133 (61.6%)	83 (38.4%)	

*BMI* Body mass index, *I-IONM* Intermittent intraoperative neural monitoring, *C-IONM* Continuous intraoperative neural monitoring, *Tx* Thyroidectomy, *CND* Central neck dissection, *ITA* Inferior thyroid artery, *NRLN* Non-recurrent laryngeal nerve, *RLN* Recurrent laryngeal nerve, *ZT* Zuckerkandl Tubercle

### The relationship between RLN and ITA

**Fig. 2:**
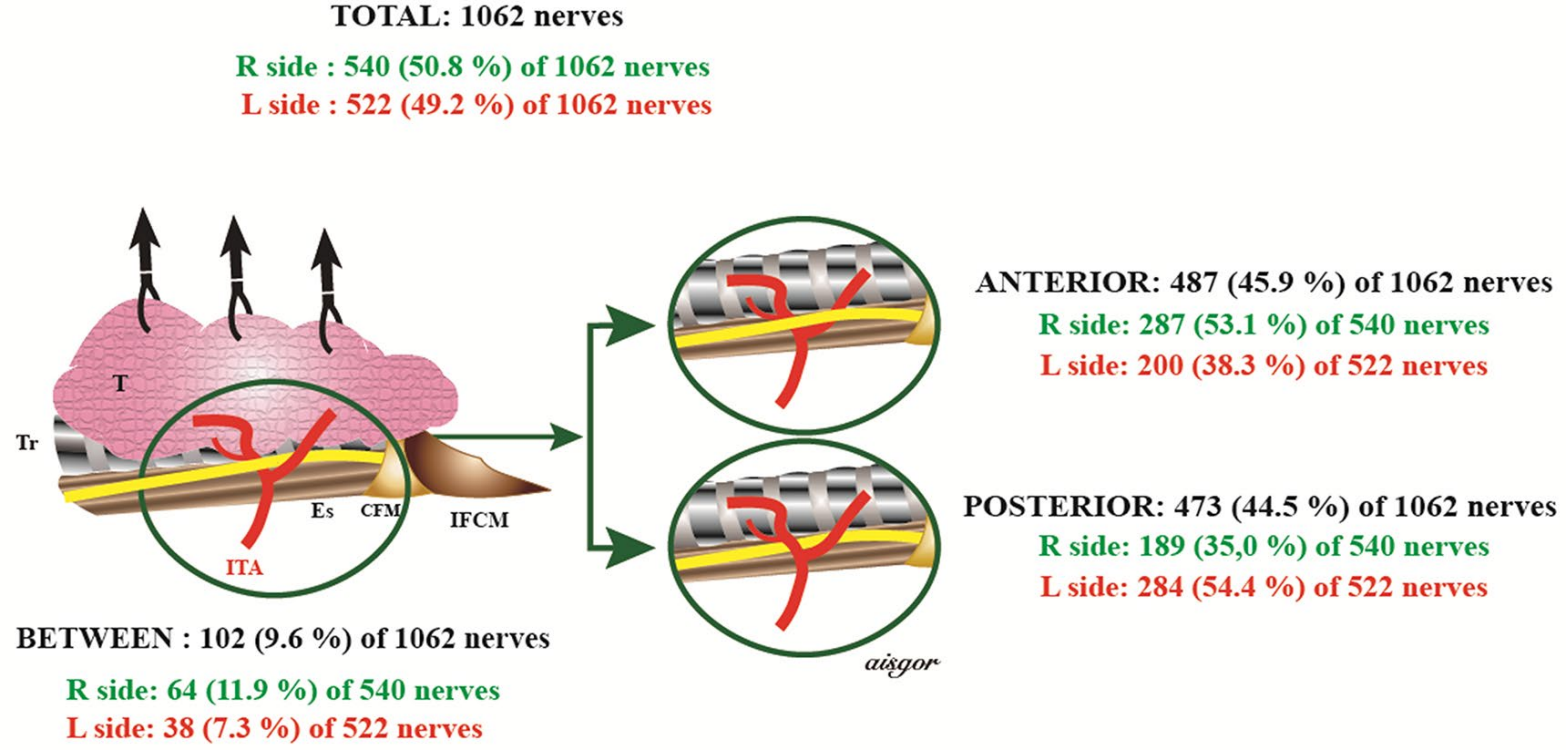
RLN-ITA relationship on the right and left side in total nerves. *R* Right, *L* Left, *T* Thyroid, *Tr* Trachea, *ITA* Inferior Thyroid Artery, *Es* Esophagus, *CFM* Cricopharyngeal Muscle, *IFCM* Inferior Pharyngeal Constrictor Muscle

**Fig. 3:**
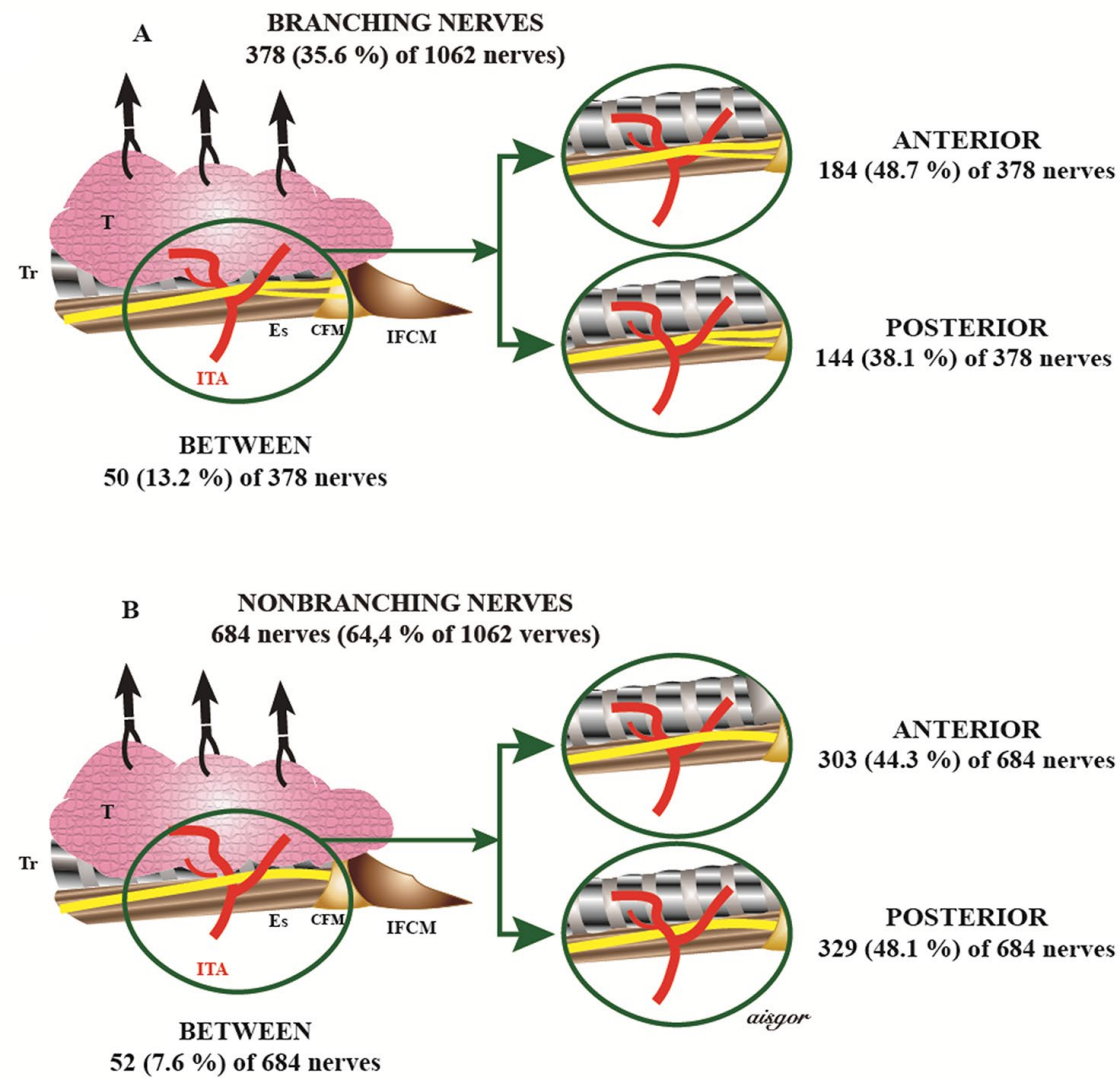
RLN-ITA relationship in branching and non-branching nerves; **A** Branching Nerves; **B** Non-Branching Nerves. *T* Thyroid, *Tr* Trachea, *ITA* Inferior Thyroid Artery, *Es* Esophagus, *CFM* Cricopharyngeal Muscle, *IFCM* Inferior Pharyngeal Constrictor Muscle. No permission is required to use the figure

RLN-ITA relationship was evaluated on 1062 neck sides, except for 2 NRLN that did not cross ITA and 6 neck sides where ITA nerve relationship was not specified (Table 4). Of the 1062 nerves; 45.9% of them were anterior to ITA, 44.5% were posterior to ITA and 9.6% were between the branches of ITA (Fig. 2). 53.1%, 35%, and 11.9%, of the RLNs on the right and 38.3%, 54.4%, and %7.3 on the left sides of the neck were anterior to ITA, posterior to ITA, between the branches of the ITA, respectively; the difference between the two neck sides was significant. 53.1% of the RLNs on the right were anterior and 54.4% of the RLNs on the left were posterior to ITA, and the difference was found significant (*p* < 0.0001). 44.3%, 48.1%, and 7.6% of the non-branching and 48.7%, 38.1%, and 13.2% of branching nerves were anterior to ITA, posterior to ITA, and between the branches of the ITA, respectively; the difference was significant (*p* = 0.001) (Fig. 3A, B). The branching distance was different regarding the relation of RLN to ITA (*p* = 0.015). The weight of the resected thyroid lobe was significantly higher in the RLNs between the branches of ITA (*p* = 0.005). No significant difference was found regarding the other properties.

**Table 4:** Comparison of the relation patterns of recurrent laryngeal nerve to inferior thyroid artery regarding the clinical and anatomical features

	Anterior RLN *n*: 487 (45.9%)	Posterior RLN *n*: 473 (44.5%)	RLN between the branches of ITA *n*: 102 (9.6%)	*P* value
Age	49 *±* 13.5	49.3 *±* 13.2	50.8 *±* 13.8	0.433
BMI kg/m^2^	28.7 *±* 5.7	29.3 *±* 6	28.4 *±* 6.2	0.185
Gender				0.995
Female (*n* = 812)	373 (45.9%)	361 (44.5%)	78 (9.6%)	
Male (*n* = 250)	114 (45.6%)	112 (44.8%)	24 (9.6%)	
Monitorisation type				0.093
I-IONM (*n* = 508)	216 (42.5%)	243 (47.8%)	49 (9.7%)	
C-IONM (*n* = 554)	271 (48.9%)	230 (41.5%)	53 (9.6%)	
Intervention type				0.058
Tx (*n* = 954)	426 (44.7%)	433 (45.4%)	95 (9.9%)	
Tx + CND (*n* = 108)	61 (56.5%)	40 (37%)	7 (6.5%)	
NRLN				0.116
(+) (*n* = 2)	1 (50%)	0	1 (50%)	
(−) (*n* = 1060)	486 (45.8%)	473 (44.6%)	101 (9.6%)	
Neck side				< 0.0001
Right (n: 540)	287 (53.1%)	189 (35%)	64 (11.9%)	
Left (n: 522)	200 (38.3%)	284 (54.4%)	38 (7.3%)	
RLN branching				0.001
Nonbranching (n: 684)	303 (44.3%)	329 (48.1%)	52 (7.6%)	
Branching (n: 378)	184 (48.7%)	144 (38.1%)	50 (13.2%)	
Branching distance (cm)	2 *±* 0.87	1.74 *±* 0.81	2.2 *±* 1.14	0.015
Weight of thyroid lobe (g)	28.2 *±* 29.9	30.2 *±* 34.2	38.4 *±* 33.2	0.005
Grade of ZT (n: 956)				0.276
Grade 0 or 1 (*n* = 566)	273 (61.5%)	246 (57.9%)	47 (54%)	
Grade 2 (*n* = 174)	83 (18.7%)	77 (18.1%)	14 (16.1%)	
Grade 3 (*n* = 216)	88 (19.8%)	102 (24%)	26 (22.6%)	

*BMI* Body mass index, *I-IONM* Intermittent intraoperative neural monitoring, *C-IONM* Continuous intraoperative neural monitoring, *Tx* Thyroidectomy, *CND* Central neck dissection, *ITA* Inferior thyroid artery, *NRLN* Nonrecurrent laryngeal nerve, *RLN* Recurrent laryngeal nerve, *ZT* Zuckerkandl tubercle

### Vocal cord paralysis

Total VCP was 4.8%, temporary VCP was 4.5%, and permanent VCP was 0.3% in the study (Table 5). Total VCP rates (6.8% vs. 3.6%, *p* = 0.018, respectively) and temporary VCP (6.8% vs. 3.2%, *p* = 0.006, respectively) were significantly higher in branching nerves compared to nonbranching nerves. Total VCP was 7.2% when RLN crossed ITA anteriorly, 2.5% when it crossed ITA posteriorly, and 2.9% when it crossed between the branches of ITA, and the difference was found significant (*p* = 0.003). Temporary VCP was 6.8% when RLN was anterior to ITA, 2.3% when it was posterior to ITA, and 2.9% when it crossed between the branches of ITA, and the difference was detected significant (*p* = 0.004). VCP rates were similar on the right and left sides even though the RLN-ITA relationship was different on the right and left sides. The rates of VCP were similar between the right and left sides in the anterior, posterior, and between the branches patterns of RLN to ITA (*p* > 0.05 for all comparisons).

**Table 5:** Comparison of the total, temporary and permanent vocal cord paralyses regarding the clinical and anatomical features

	Total VCP	*p*	OR 95% CI	Temporary VCP	*p*	OR 95% CI	Permanent VCP	*p*	OR 95% CI
Age		0.659	1.003 0.983–1.024		0.996	0.999 0.978–1.021		0.078	1.063 0.976–1.158
VCP (n: 51)	49.8 *±* 11.8			49.1 *±* 11.8			60.3 *±* 4.9		
VCN (n: 1019)	49.2 *±* 13.5			49.2 *±* 13.5			49.2 *±* 13.4		
Gender		0.990	0.996 0.513–1.933		0.796	0.916 0.469–1.788		1	1.004 1–1.008
Female (n: 819)	39 (4.8%)			36 (4.4%)			3 (0.4%)		
Male (n: 251)	12 (4.8%)			12 (4.8%)			0		
BMI		0.752	0.999 0.949–1.053		0.903	0.996 0.944–1.052		0.464	1.036 0.869–1.234
VCP	28.9 *±* 5			28.8 *±* 5.2			30.2 *±* 1.3		
VCN	28.9 *±* 5.4			28.9 *±* 5.9			28.9 *±* 5.9		
Postoperative diagnosis		0.870			0.925			0.845	
MNG (n: 632)	31 (4.9%)		1 (Ref)	29 (4.6%)		1 (Ref)	2 (0.3%)		1 (Ref)
Graves (n: 107)	4 (3.7%)	0.600	0.753 0.260–2.178	4 (3.7%)	0.694	0.807 0.278–2.345	0	0.997	0
Malignant (n: 331)	16 (4.8%)	0.961	0.985 0.531–1.828	15 (4.5%)	0.968	0.987 0.521–1.868	1 (0.3%)	0.970	0.955 0.086–10.566
Hyperthyroidism		0.428	0.743 0.357–1.55		0.562	0.804 0.384–1.684		0.355	0.996 0.992–1.000
(+) (n: 237)	9 (3.8%)			9 (3.8%)			0		
(−) (n: 833)	42 (5%)			39 (4.7%)			3 (0.4%)		
Neck side		0.995	0.998 0.569–1.752		0.871	0.953 0.534–1.701		0.618	2.080 0.188–23.011
Right (n: 545)	26 (4.8%)			25 (4.6%)			1 (0.2%)		
Left (n: 525)	25 (4.8%)			23 (4.4%)			2 (0.4%)		
Monitorisation type		0.178	0.678 0.384–1.196		0.070	0.583 0.322–1.052		0.251	1.005 0.999–1.012
I-IONM (n: 510)	29 (5.7%)			29 (5.7%)			0		
C-IONM (n: 560)	22 (3.9)%			19 (3.4%)			3 (0.5.%)		
Intervention type		0.421	1.4 0.615–3.187		0.621	1.248 0.518–3.005		0.192	4.350 0.391–48.363
Tx (n: 959)	44 (4.6%)			42 (4.4%)			2 (0.2%)		
Tx + CND (n: 111)	7 (6.3%)			6 (5.4%)			1 (0.9%)		
NRLN		0.178	6.773 0.692–66.281		0.168	7.227 0.738–70.796		1	0.997 0.994–1.000
(+) (n: 4)	1 (25%)			1 (25%)			0		
(−) (n: 1066)	50 (4.7%)			47 (4.4%)			3 (0.3%)		
Relation of RLN to ITA (n: 1062)		0.002	*		0.003	**		0.863	
Anterior^1^ (n: 487)	35 (7.2%)		1 (Ref)	33 (6.8%)	Ref.	1 (Ref)	2 (0.4%)		1 (Ref)
Posterior^2^ (n: 473)	12 (2.5%)	0.001	0.336 0.172–0.656	11 (2.3%)	0.001	0.328 0.164–0.656	1 (0.2%)	0.587	0.514 0.046–5.685
Crossing^3^ (n: 102)	3 (2.9%)	0.125	0.391 0.118–1.298	3 (2.9%)	0.175	0.417 0.125–1387	0	0.997	0
Branching type		0.018			0.006			0.556	0.996 0.991–1.001
Nonbranching (n: 690)	25 (3.6%)		1 (reference)	22 (3.2%)		1 (reference)	3 (0.4%)		
Branching (n: 380)	26 (6.8%)		1.954 1.112–3.434	26 (6.8%)		2.230 1.246–3.992	0		
Branching distance (cm)		0.126			0.126				
VCP	2.31 *±* 1.24			2.31 *±* 1.24					
VCN	1.89 *±* 0.86			1.89 *±* 0.86					
Grade of ZT (n: 960)		0.394			0.587			0.357	
Grade 0 or 1 (n: 570)	29 (5.1%)		1 (Ref)	26 (4.6%)		1 (Ref)	3 (0.5%)		1 (Ref)
Grade 2 (n: 174)	5 (2.9)%	0.227	0.552 0.210–1.448	5 (2.9%)	0.334	0.619 0.234–1.637	0	0.996	0
Grade 3 (n: 216)	8 (3.7%)	0.415	0.718 0.323–1.595	8 (3.7%)	0.598	0.805 0.359–1.806	0	0.995	0
Weight of thyroid lobe (g)		0.633	1.002 0.993–1012		0.744	1.002 0.992–1.012		0.578	1.007 0.983–1.032
VCP	32.7 *±* 24.3			32 *±* 21.1			40.7 *±* 55.8		
VCN	30.1 *±* 32.7			30.1 *±* 32.1			30.1 *±* 32.3		

*VCP* Vocal cord paralysis, *VCN* Normal vocal cord function, *OR* Odds ratio, *CI* Confidence interval, *MNG* Multinodular goiter, *BMI* Body mass index, *I-IONM* Intermittent intraoperative neural monitoring, *C-IONM* Continuous intraoperative neural monitoring, *Tx* Thyroidectomy, *CND* Central neck dissection, *ITA* Inferior thyroid artery, *NRLN* Nonrecurrent laryngeal nerve, *RLN* Recurrent laryngeal nerve, *ZT* Zuckerkandl tubercle, *Ref* Reference

*1 vs. 2 *p* = 0.001 1 vs. 3 *p* = 0.125, 2 vs. 3, *p* = 0.737 (OR: 0.859, 95% CI: 0.238–3.101)

**1 vs. 2 *p* = 0.001, 1 vs. 3 *p* = 0.175, 2 vs. 3, *p* = 0.786 (OR: 0.859, 95% CI: 0.215–2.868)

Both RLN branching and RLN crossing the artery anteriorly were determined as independent risk factors for total and temporary VCP in logistic regression analysis. Nerve branching increases total VCP approximately by 2-fold and temporary VCP by 2.2-fold, compared to the nonbranching nerves. RLN crossing the ITA anteriorly increases total VCP 2.8 times (*p* = 0.002), temporary VCP 2.9 times (*p* = 0.003), compared to RLN crossing the ITA posteriorly (Table 6).

**Table 6:** Multivariable logistic regression analyses of risk factors for postoperative total and temporary vocal cord paralysis

	VCP			
	Total VCP		Temporary VCP	
	Odds ratio	*p*	Odds ratio	*p*
RLN ITA relation				
Posterior to ITA	0.353 (0.180–0.690)	0.002	0.337 (0.173–0.696)	0.003
Between the branches of ITA	0.361 (0.108–1.202)	0.097	0.378 (0.113–1.264)	0.114
Anterior to ITA	1.00 (reference)		1.00 (reference)	
RLN branching				
Branching	1.958 (1.101–3.481)	0.002	2.245 (1.238–4.070)	0.008
Non-branching	1.00 (reference)		1.00 (reference)	

*VCP* Vocal cord paralysis, *RLN* Recurrent laryngeal nerve, *ITA* Inferior thyroid artery

## Discussion

Anatomical variations of RLN are frequent and variable and cannot be predicted preoperatively. There is a very few number of studies evaluating the relationship between anatomical variations of RLN and VCP, even though the risk of VCP has been evaluated in many studies. Our study determined both the extralaryngeal branching and RLN crossing the ITA from the anterior as independent risk factors for increase in total and temporary VCP. In the case of RLN crossing the ITA anteriorly, the rates of both total RLN (7.4% vs. 2.6% vs. 2.8%, respectively; *p* = 0.001) and temporary RLN paralysis (7.1% vs. 2.4% vs. 2.8%, respectively; *p* = 0.001) were significantly higher compared to those when the RLN was posterior to ITA and between the branches of ITA. This is the first study in the literature demonstrating that the relationship between RLN and ITA has an impact on the risk of VCP. The rate of the total (6.8% vs. 3.8% respectively; *p* = 0.028) and temporary (6.8% vs. 3.4% respectively; *p* = 0.011) VCP was higher in extralaryngeal branching nerves compared to non-branching nerves. There were no significant differences in permanent paralysis in terms of both nerve artery relationship and nerve branching. There was no significant difference in total, temporary, permanent VCP in NRLNs according to ITA.

The relationship between RLN and ITA has been evaluated in many studies. Henry et al. evaluated the relationship between RLN and ITA and found that 27.6% (95% CI:23.2–30.6) of 14,269 RLN crossed the ITA anteriorly (95% CI: 45.2–53.5), 50.7% (95% CI:45.2–53.5) posteriorly, 21.7% (95% CI:17.8–24.6) between the branches of ITA in a meta-analysis involving 79 studies (18 intraoperative cases, 60 cadavers, 1 intraoperative + cadaver case). However, 62.6% (95% CI:56.3–65.7) of the nerves on the left side were in the posterior position and only 37% (95% CI:45.2–53.5) on the right side were in the posterior position; the difference was significant. Subgroup analysis revealed no significant difference in study type, gender, and geographic region [13]. However, both right (65%) and left (45%) RLNs were reported to be in the posterior of ITA in a systematic review by Ling et al. [18]. In our study, of the RLNs on the right and left sides; 53.1%, 38.3%, was anterior and 35%, 54.4% was posterior to the ITA, and 11.9%, 7.3% was between the branches of the ITA, respectively, with a significant difference (Table 4; Fig. 2). However, there were no significant differences between the right and left sides in terms of VCP. In the last cadaver study, RLN was found to be in the anterior and posterior positions with the rates of 68%, 32% on the right, and 32%, 68% on the left, respectively [19]. Left RLN has been reported to give more branches to the esophagus and trachea in Gray’s Anatomy [20]. It has been suggested that this is an anatomical feature that may explain the RLN being more posterior on the left side [13].

This is the first study showing that the relationship between RLN and ITA is an anatomical factor significantly affecting RLN injury. RLN crossing ITA from anterior increases total VCP by 2.8 times and temporary VCP by 2.9 times compared to posterior crossing according to the results of the study (Table 6). There is only one study evaluating VCP rates according to the relationship between RLN and ITA in the literature. Sancho et al. found VCP rates as 15% when RLN was anterior to ITA, 14.7% when it was posterior to ITA, and 9.1% when it was between the branches of ITA, and did not find any significant difference in terms of VCP according to the position of RLN, contrary to our results (*p* = 0.529). The rates of VCP in this study were higher than the present study, however, the use of IONM in the current study might have affected the difference between the two studies [12].

In current literature, some specific types regarding the anatomical relationship between ITA and RLN have been reported to increase the risk of RLN. However, these data are based on anatomical studies or pre-IONM studies. Fowler and Hanson and Skandalakis argued that the risk of injury may be higher when RLN crosses ITA anteriorly or between the branches of ITA in their anatomy studies [14]. Skandalakis et al. argued that VCP may occur due to the stretching of the nerve during the traction of the thyroid lobe, when RLN is in the ITA anterior position. They argued that careless dividing of ITA may increase the risk of injury if RLN is between the branches of the artery [15].

However, our results do not support the claim that between the branches of ITA pattern increases the risk of RLN injury. Although no significant difference was detected, the risk of injury was less than half in cases with RLN crossing between the branches of ITA compared to the anterior crossing pattern of RLN (OR: 0.361; *p* = 0.097 for total VCP, OR: 0.378; *p* = 0.114 for temporary VCP) (Table 6). Yalcin et al. stated that a particularly high-risk situation would arise when the ITA splits into many branches close to the RLN and the branches of both structures are intertwined [21]. Sometimes nerve injury may be caused by clamping or electrocauterization to stop bleeding of the ITA’s branches close to the RLN [22]. Before IONM, the most common etiological factors for VCP due to the RLN injury were thought to be nerve cutting or clamping.However, IONM studies revealed that traction trauma has been the most common etiological factor [23].

Extralaryngeal branching of RLN is common and may be bilateral or unilateral [24]. The rate of nerve branching was 35.6% and the branching distance was 1.92 *±* 0.9 cm in our study (Table 2; Fig. 3A). The majority of branching nerves (92.4%) had 2 branches, 6.8% 3 branches, and rarely (0.8%) 4 branches. The rate of nerve branching was higher in females compared to males (38.5%, 25.8%, *p* < 0.0001, respectively). The branching rate was approximately 50% when RLN crossed between the branches of ITA, 38% when it crossed from the anterior, and 30% when it crossed from the posterior; the difference was significant. Fontonet et al. found no difference between male and female gender in terms of branching rate but found that the branching distance was higher in females compared to males. Accordingly, they suggested that the risk of nerve injury was higher and the fact that the visual identification and protection of the nerves was difficult due to the thinner extralaryngeal branches in the nerves supported the idea of easy injury [25]. In some studies, branching on the right side was more common compared to the left side [12]. Extralaryngeal branching was approximately 40% in intraoperative studies and 73% in cadaver series in the meta-analysis involving more than 28,000 RLNs. It has been reported that the nerve oftenly divided into 2 branches at the most distal 2 cm [26]. In the cadaver series, the number of extralaryngeal branches is also higher such as the branching rate, and the number of more than 2 branches is over 40% [19]. This suggests that both the branching rate and the number of branches are underreported.

Logistic regression analysis revealed that the risk of total VCP increased approximately by 2-fold and the risk of temporary VCP increased by 2.2-fold in branching nerves in the present study (Table 6). Barczynski et al. also found that the rate of temporary VCP was higher in branching nerves and reported that the risk of paralysis increased 2.98 times in branching nerves (95% CI 1.79–4.95; *p* = 0.001). However, they found no difference in terms of the permanent VCP rate between branching and non-branching nerves (1.1% vs. 0.2%, respectively) [27]. Casella et al. reported that branching nerves were more frequently associated with VCP than non-branching nerves, and that the risk of unilateral temporary VCP was 7.36 times higher (95%CI: 1.84–29.4; *p* = 0.0061) and the risk of unilateral permanent paralysis was 13.25 times (95%CI: 1.42–123.73; *p* = 0.0204) higher [11]. Sancho et al. similarly found that the rate of VCP was higher in branching nerves (15.8% vs. 8.1%, respectively *p* = 0.022) and the risk of VCP was 2.2 times higher (95%CI: 1.1–4.5). In addition, they reported that the branching distance in branching nerves with VCP was longer compared to the branching nerves without VCP (29.4 + 10.4 vs. 19.1 + 9.8, respectively, *p* = 0.003) [12].

We found no significant difference between the branching nerves with VCP and normal vocal cord function regarding the branching distance. Fontonet et al. determined that the branching distance was longer in females compared to males (11.04 *±* 0.60 mm vs. 8.55 *±* 0.76 mm, respectively, *p* = 0.012) and suggested that the risk of nerve injury was higher accordingly. In addition, they claimed that the fact that visual identification and protection of the nerves was difficult due to the thinner extralaryngeal branches in the branching nerves supported the idea of easy injury [25]. In some other studies, VCP rates were found to be similar in branching and non-branching nerves [9, 10, 24].

RLN is fixed at two points between the first part of the subclavian artery on the right or around the arcus aorta on the left and the laryngeal entrance under the cricopharyngeal muscle. In thyroidectomy, when the thyroid lobe is tractioned anteromedially, a compressive force occurs between these two fixed points of the nerve [6]. We believe that RLN, especially coursing anterior to the ITA, may be exposed to a greater risk of RLN injury for two possible reasons. With anteromedial traction of the thyroid lobe, more traction force may be reflected to the nerve by being exposed to more elevation and more pronounced artificial angulation. When the nerve is tractioned laterally, the maximal tension is located at the angulation zone and the distal 2 cm [28]. In addition, the effect of the same traction force on the branching nerves is reflected more since the diameter of the branching nerves is less than the non-branching nerves, since the traction power reflected upon the nerve is inversely proportional to the square of the diameter of the nerve. The same traction force causes more tensile stress on thin nerves [29]. The risk of injury may be higher, especially both in the nerves crossing ITA anteriorly and branching. More caution in thyroid lobe traction, especially in the case of anterior positioning and/or branching of RLN, and fully exposing the RLN and defining its anatomical variations may reduce the risk of VCP.

Another factor is that while the branches of ITA are divided by energy devices close to the thyroid capsule, the nerve in the anterior position may be more affected by lateral heat dissipation. For this reason, the energy device should be applied at a safe distance on the thyroid capsule considering the RLN’s course and position.

One of the main limitations of our study is that it is retrospective. Another is that the course of RLN in the thyroid region ((normal trajectory, abnormal acquired-medial, abnormal acquired-ventral), fixed, splayed or entrapped, and posterior ligament of Berry or associated vessel neural entrapment), which have been recently defined in the international anatomical classification system of RLN, have not been evaluated [30]. However, it is one of the largest studies evaluating anatomical variations of RLN and VCP, and it is the first study demonstrating the effect of the relationship between RLN and ITA on VCP.

## Conclusion

As a result, among the variables evaluated in the study, extralaryngeal RLN branching related to RLN anatomy and the anatomical relationship between ITA RLN were identified as potential independent risk factors for postoperative VCP.

RLN crossing ITA anteriorly and RLN branching are frequent anatomical variations increasing the risk of VCP in thyroidectomy, that cannot be predicted preoperatively. Therefore, exposing the entire RLN course and determining its anatomical variations may be an important risk reduction strategy.

## Data Availability

No datasets were generated or analysed during the current study.
